# Symbiotic microalgal diversity within lichenicolous lichens and crustose hosts on Iberian Peninsula gypsum biocrusts

**DOI:** 10.1038/s41598-020-71046-2

**Published:** 2020-08-20

**Authors:** Patricia Moya, Arantzazu Molins, Salvador Chiva, Joaquín Bastida, Eva Barreno

**Affiliations:** 1 Botánica, ICBIBE, Fac. CC. Biológicas, Universitat de València, C/ Dr. Moliner, 50, 46100 Burjassot, Valencia Spain; 2 Geología, Fac. CC. Biológicas, Universitat de València, C/ Dr. Moliner, 50, 46100 Burjassot, Valencia Spain

**Keywords:** Microbiology, Plant sciences

## Abstract

This study analyses the interactions among crustose and lichenicolous lichens growing on gypsum biocrusts. The selected community was composed of *Acarospora nodulosa*, *Acarospora placodiiformis*, *Diploschistes diacapsis*, *Rhizocarpon malenconianum* and *Diplotomma rivas-martinezii*. These species represent an optimal system for investigating the strategies used to share phycobionts because *Acarospora* spp*.* are parasites of *D. diacapsis* during their first growth stages, while in mature stages, they can develop independently. *R. malenconianum* is an obligate lichenicolous lichen on *D. diacapsis*, and *D. rivas-martinezii* occurs physically close to *D. diacapsis*. Microalgal diversity was studied by Sanger sequencing and 454-pyrosequencing of the nrITS region, and the microalgae were characterized ultrastructurally. Mycobionts were studied by performing phylogenetic analyses. Mineralogical and macro- and micro-element patterns were analysed to evaluate their influence on the microalgal pool available in the substrate. The intrathalline coexistence of various microalgal lineages was confirmed in all mycobionts. *D. diacapsis* was confirmed as an algal donor, and the associated lichenicolous lichens acquired their phycobionts in two ways: maintenance of the hosts’ microalgae and algal switching. Fe and Sr were the most abundant microelements in the substrates but no significant relationship was found with the microalgal diversity. The range of associated phycobionts are influenced by thallus morphology.

## Introduction

Lichens are a well-known and reasonably well-studied examples of obligate fungal symbiosis^1,2^. They have traditionally been considered the symbiotic phenotype resulting from the interactions of a single fungal partner and one or a few photosynthetic partners. However, lichen symbiosis has been shown to be far more complex and may include a wide range of other interacting organisms, including nonphotosynthetic bacteria, accessory fungi, and algae^3,4^.

Biological soil crusts (BSCs; biocrusts) are complex communities of multiple organisms, including lichens, which live on the soil surface, creating a consistent layer and binding soil particles due to their architecture and activity^5^. Biocrusts are present in a wide variety of ecosystems; however, they are most abundant in harsh environments^6^ with sparse vascular vegetation cover, where they are considered pioneers in colonizing free soil surfaces^7^. Biocrusts dominated by lichens have a high conservation value due to their potential to form extended covers, greatly contributing to ecosystems considered hotspots of biodiversity^8^.

Lichen biocrusts are particularly notable in gypsum ecosystems^9,10^. Some areas distributed throughout the central Iberian Peninsula show characteristic gypsum outcrops colonized by a predominant group of crustose lichen species, i.e., *D. diacapsis* (Ach.) Lumbsch, *Acarospora placodiiformis* H. Magn., *Acarospora nodulosa* (Dufour) Hu*.*, and occasionally *D. rivas-martinezii* (Barreno et A. Crespo) Barreno et al.^11^ and *Rhizocarpon malenconianum* (Llimona et Werner) Hafellner et Mayrhofer^12^. Meanwhile, *A. placodiiformis*, *D. rivas-martinezii* and *R. malenconianum* occur exclusively on gypsum soils, *A. nodulosa* and *D. diacapsis* are preferential gypsophytes, i.e., species with a strong preference for gypsum soils, but are also occasionally found outside these substrata^13,14^. Moreover, crustose lichens occurring in these communities show peculiar lifestyles^13^ as facultative or obligate lichenicolous lichens: lichenized fungi that form their own mutualistic and photosynthetic thalli within or on their hosts^15–19^. In these communities, *Acarospora* spp*.* are parasites of *D. diacapsis* during their first growth stages, while in mature stages, they can develop independently. *R. malenconianum* is an obligate lichenicolous lichen on *D. diacapsis*, and the epilithic species *D. rivas*-*martinezii* is regularly found physically close to *D. diacapsis*.

These lichenicolous lichens take advantage of the microalgae available in the host’s thallus, thus avoiding wasting energy and trying to find an appropriate algal partner in the substrate^20^. The lichenicolous lichen *Diploschistes muscorum* growing on *Cladonia* spp.^20,21^ has been traditionally studied to understand the way in which phycobionts are shared in these parasitic lichens. Friedl^21^ found that *Diploschistes* starts as a lichenicolous fungus, invading the *Cladonia* thallus and utilizing the alga at an intermediate stage. Then, *Diploschistes* switches the *Cladonia* phycobiont (*Asterochloris* sp.) for a more suitable microalga (*Trebouxia* sp.), with which it forms an independent thallus. Hence, algal switching was established as the usual strategy in lichenicolous lichens, and then it was reported for lichen communities where algal morphospecies and genotypes are shared among different fungal genera and families^22^. Lücking and Grube^23^ also found switching to different microalgae in lichenicolous morphs of the foliicolous species *Chroodiscus coccineus*. However, other authors did not find this switching strategy, but rather the maintenance of the hosts’ microalgae: for example, *Paralecanographa grumulosa* infests *Roccella* thalli^24^, *Blarneya hibernica*, parasitizes species of *Enterographa* and *Lecanactis*^25^, *Gyalolechia bracteata* grows as a parasite on *Thalloidima sedifolium*^26,27^, and *Rimularia insularis* colonizes *Lecanora rupicola*^28^.

Recently, lichen-associated microbiomes and phycobionts in the parasite *Diploschistes muscorum* and its host *Cladonia symphycarpa* were studied in depth by Wedin et al.^20^. The authors concluded that some parasitic lichens may acquire their compatible phycobionts by stealing them from the host lichen, explained as the maintenance of the hosts’ algae, while others associate with a different algal partner, termed algal switching^22^, or host multiple algal types (coexistence). Thus, questions related to the strategies used to acquire phycobionts in lichenicolous lichens remain open. Crustose gypsum lichenized fungi and associated lichenicolous lichens represent an optimal system for investigating such strategies, which also challenge concepts such as selectivity or specificity. As mentioned in Muggia et al.^29^, ‘specificity’ was initially defined as the possible taxonomic range of acceptable partners, in contrast to ‘selectivity’, which indicated the frequency of associations between compatible partners^30–33^. Currently, ‘selectivity’ and ‘specificity’ may also be regarded as multidirectional in terms of characterizing relationships among lichen symbionts.

The presence of multiple phycobiont species (coexistence) within a single lichen thallus is a relatively common phenomenon in lichen symbioses. The coexistence of multiple *Trebouxia* species within a single lichen thallus was studied in depth in the epiphytic species *Ramalina farinacea* from the Mediterranean region^34–36^ and in other lichens with diverse geographic origins and growth forms^37–45^. However, it was not analysed in depth in crustose lichen species^46–48^, and in the specific case of gypsum crustose parasitic lichens, little information is available related to algal diversity.

This work is complementary to that of Moya et al.^49,50^, where microalgal diversity was analysed in foliose and dimorphic *Cladonia* spp*.* and in squamulose *Psora decipiens*, *P. saviczii*, *Clavascidium* spp. and *Placidium* spp. developed on the same central Iberian Peninsula Miocene gypsum biocrusts. Moya et al.^49^ provided a detailed characterization of a novel phycobiont species (*Asterochloris mediterranea*) detected in the thalli of *Cladonia* spp., and Moya et al.^50^ proved that *Myrmecia israeliensis* was the primary symbiotic microalga in squamulose lichens located in these BSCs. For this investigation, we deal with the crustose community, and this complementarity allows us to overview the symbiotic patterns in the whole lichen community.

In this study, we analysed microalgal diversity and interaction patterns in crustose lichenized and lichenicolous lichens on gypsum by Sanger sequencing and 454-pyrosequencing of the nrITS (internal transcribed spacer) region and characterized the microalgae ultrastructurally. Mycobionts were studied by performing phylogenetic analyses. Moreover, the geochemical characteristics of these peculiar gypsum outcrops that are likely to influence the presence of specific lichen species or microalgal availability in the substrate are still unknown^13^. However, Cania et al.^51^ recently described interactions between substrate characteristics and microbial communities and found that different soil substrates harbour distinct bacterial groups. To characterize the mineralogical compositions of those gypsum soils, spectrometric techniques were applied to soil samples collected from each studied location, and macro- and micro-element patterns were analysed to evaluate their influence on the microalgal pool available in the substrata.

In addition, both lichens and their phycobionts perform essential functions in terrestrial ecosystems, but because they are often not well known in different types of habitats, they are underrepresented in conservation assessments and implementation when compared to other groups of organisms, such as vascular plants or animals^52^. These results could be incorporated into frameworks to protect the extraordinary biodiversity hosted by Miocene gypsum outcrops in the Iberian Peninsula.

## Results

### Sequencing success

**Figure 1:**
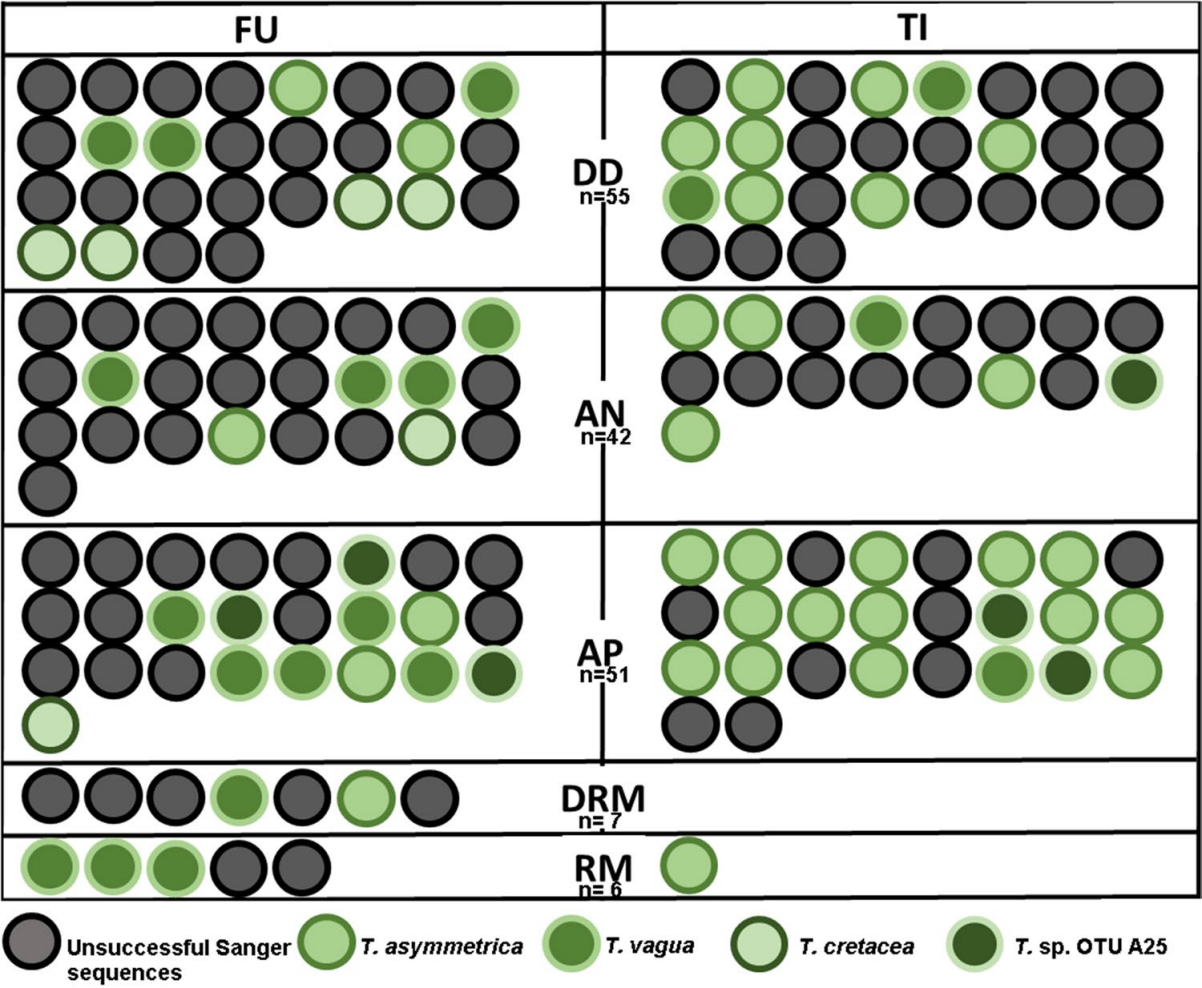
Sanger sequencing success and *Trebouxia* diversity detected in *Diploschistes diacapsis* (DD), *Acarospora nodulosa* (AN), *Acarospora placodiiformis* (AP), *Rhizocarpon malenconianum* (RM) and *Diplotomma rivas-martinezii* (DRM) collected in Fuentidueña de Tajo (FU) and Titulcia (TI). The total number of specimens analysed for each species is indicated (n). Sequences passing the quality filter were considered unique, showing only one predominant phycobiont (green circles). Sequences with ambiguous base calls and/or underlying peaks in the electrophoretogram were excluded from the dataset and noted as unsuccessful Sanger sequences (grey circles).

From a total of 55 *D. diacapsis* (DD) specimens analysed, 37 showed unsuccessful Sanger sequences, and only 18 showed unique sequences. The ratios of unsuccessful Sanger sequences versus unique sequences in the remaining lichen species are shown in Fig. 1: 30 versus 12 in *A. nodulosa* (AN), 23 versus 28 in *A. placodiiformis* (AP), 2 versus 4 in *R. malenconianum* (RM) and 5 versus 2 in *D. rivas*-*martinezii* (DRM). All the species selected showed unsuccessful Sanger sequence frequencies ranging from 33% in RM to 71% in the cases of AN and DRM.

### Phycobiont diversity detected by Sanger sequencing (nrITS)

**Figure 2:**
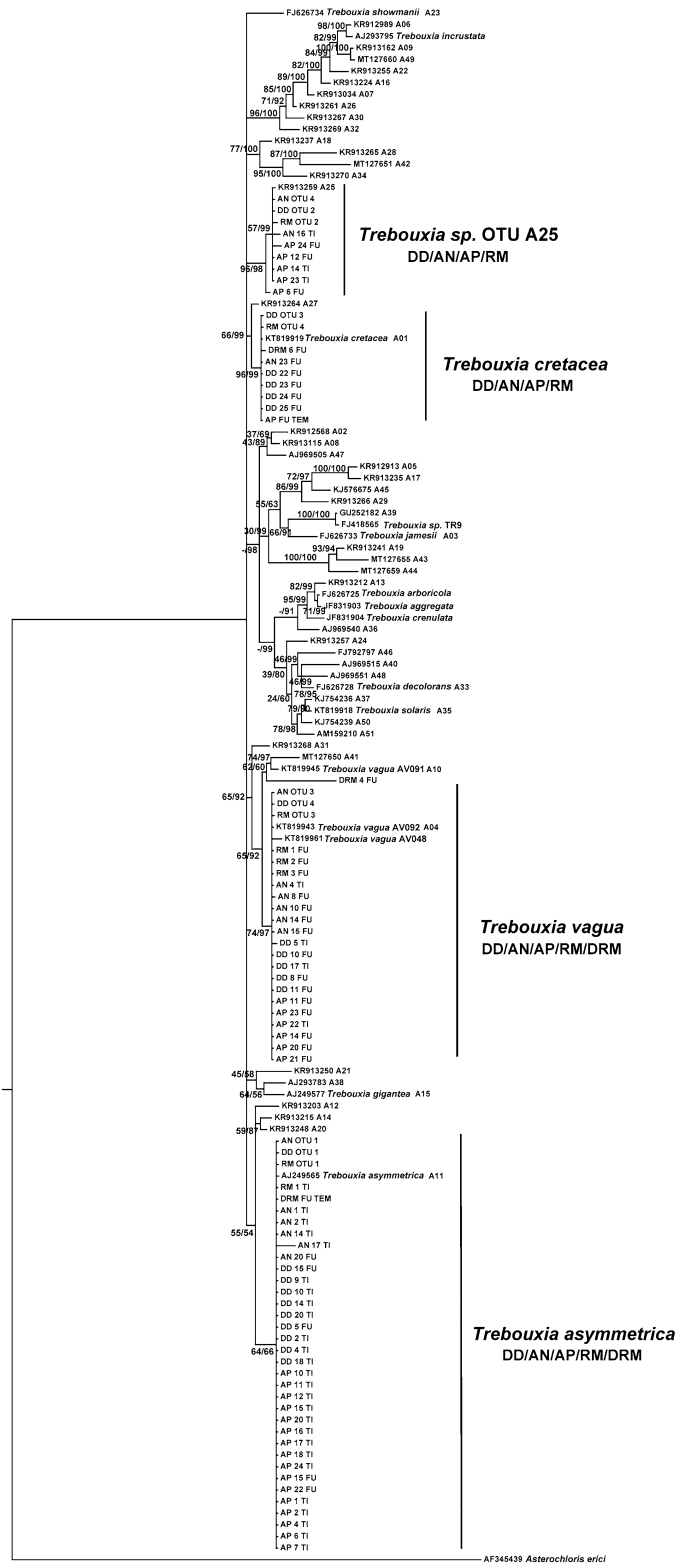
*Trebouxia* diversity revealed by Sanger sequencing and 454-pyrosequencing of *Diploschistes diacapsis* (DD), *Acarospora nodulosa* (AN), *Acarospora placodiiformis* (AP), *Rhizocarpon malenconianum* (RM) and *Diplotomma rivas-martinezii* (DRM). Rooted nrITS gene tree representing 134 *Trebouxia* sequences, including 57 OTUs from clade A described by Muggia et al.^110^ and retrieved from GenBank. Pyrosequencing consensus sequences were labelled with the lichen species and code_number of the OTU. The sequence obtained from the specimen selected to characterize the ultrastructure with TEM is indicated. Values at nodes indicate statistical support estimated by two methods: bootstrap support (BS, RAxML analysis) and posterior probabilities (PP, MrBayes analysis). The scale bar shows the estimated number of substitutions per site.

Among the *Trebouxia* phycobionts detected by Sanger sequencing belonging to the clade ‘A’ *arboricola/gigantea* type (Fig. 2), 32 from DD/AN/AP/RM/DRM were allocated to *Trebouxia asymmetrica* (bootstrap support (BS) value of 64/posterior probability (PP) score of 66); 20 detected in the same five lichen species matched *Trebouxia vagua* (74 BS/97 PP); 6 detected in DD/AN/AP matched *Trebouxia cretacea* (96 BS/99 PP); and six from AN/AP matched *Trebouxia* sp. OTU A25^53^ (96 BS/98 PP). *T. asymmetrica*, *T. vagua* and *Trebouxia* sp. OTU A25 appeared in both locations (FU and TI), whereas *T. cretacea* was detected only in FU (Fig. 2).

**Figure 3:**
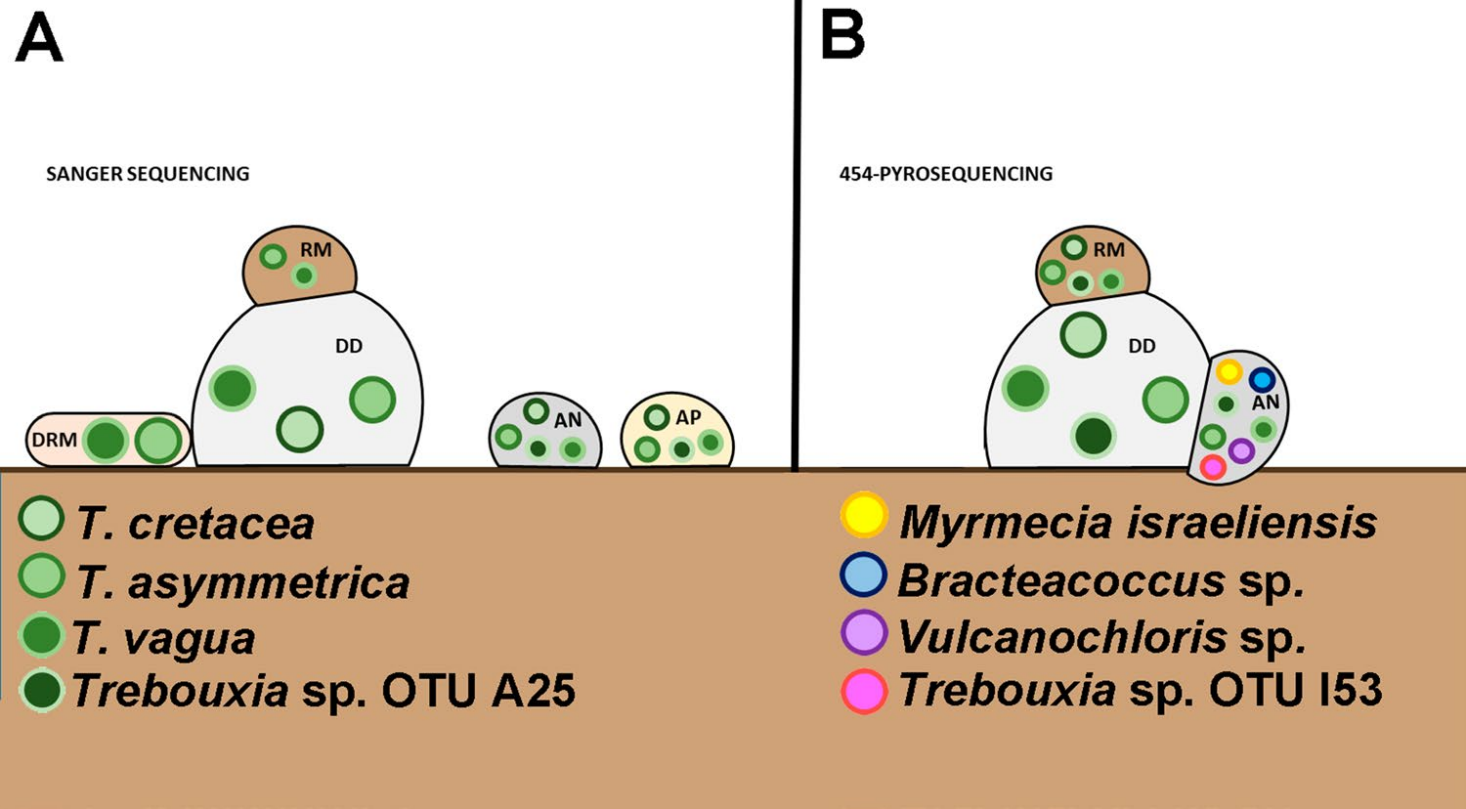
(**A**) Summary of the *Trebouxia* phycobiont diversity detected by Sanger sequencing in *Diploschistes diacapsis* (DD), *Acarospora nodulosa* (AN), *A. placodiiformis* (AP), *Rhizocarpon malenconianum* (RM) and *Diplotomma rivas-martinezii* (DRM). A total of four *Trebouxia* spp. were detected, namely, *T. asymmetrica*, *T. vagua*, *T. cretacea* and *Trebouxia* sp. OTU A25, each of which is shown as a different green circle. (**B**) Depiction of the microalgae detected by 454-pyrosequencing in three specimens of *Diploschistes diacapsis* (DD), *Acarospora nodulosa* (AN) and *Rhizocarpon malenconianum* (RM). Each microalgal strain detected is indicated with a coloured circle.

We identified three *Trebouxia* species in the thalli of DD by Sanger sequencing: *T. asymmetrica*, *T. vagua*, and *T. cretacea* (Fig. 3A). The obligate lichenicolous lichen RM hosted *T. asymmetrica* and *T. vagua*. Independent thalli of AN and AP were associated with the abovementioned three *Trebouxia* spp. and with *Trebouxia* sp. OTU A25. Epilithic DRM contained *T. asymmetrica* and *T. vagua*.

### Phycobiont diversity detected by 454-pyrosequencing

454-pyrosequencing of nrITS amplicons produced 6,218 sequence reads for DD, 4,290 for AN and 5,534 for RM. *Trebouxia asymmetrica*, *Trebouxia vagua*, *Trebouxia cretacea* and *Trebouxia* sp. OTU A25 were detected in DD (Table 1, Figs. 2, 3B). The obligate lichenicolous lichen RM hosted the same four *Trebouxia* species. AN in the parasitic state (Fig. 3B) harboured *T. asymmetrica*, *T. vagua*, *Trebouxia* sp. OTU A25, *Myrmecia israeliensis* and several minor chlorophytes.

**Table 1:** Taxonomic identification and summary of number of sequences obtained by pyrosequencing of *Trebouxia* spp., *Myrmecia* and other minor chlorophytes for each particular OTU in the three analyzed thalli, *Diploschistes diacapsis* (DD), *Acarospora nodulosa* (AN) and *Rhizocarpon malenconianum* (RM).

	DD	AN	RM
*Trebouxia asymmetrica*	OTU1_4111 (66%)	OTU1_3027 (70%)	OTU1_4972 (89%)
*Trebouxia* sp. OTU A25	OTU2_2008 (32%)	OTU4_3	OTU2_370 (6.6%)
*Trebouxia cretacea*	OTU3_83		OTU3_181
*Trebouxia vagua*	OTU4_16	OTU3_6	OTU4_11
*Trebouxia* sp. OTU I53		OTU7_2	
*Myrmecia israeliensis*		OTU2_1247 (30%)	
*Bracteacoccus*		OTU5_3	
*Vulcanochloris*		OTU6_2	

The consensus sequences of the OTUs were encoded: number of OTU_number of sequences found for this OTU. Percentage of OTU1 and OTU2 were indicate in parenthesis.

### Ultrastructural characterization of microalgae

**Figure 4:**
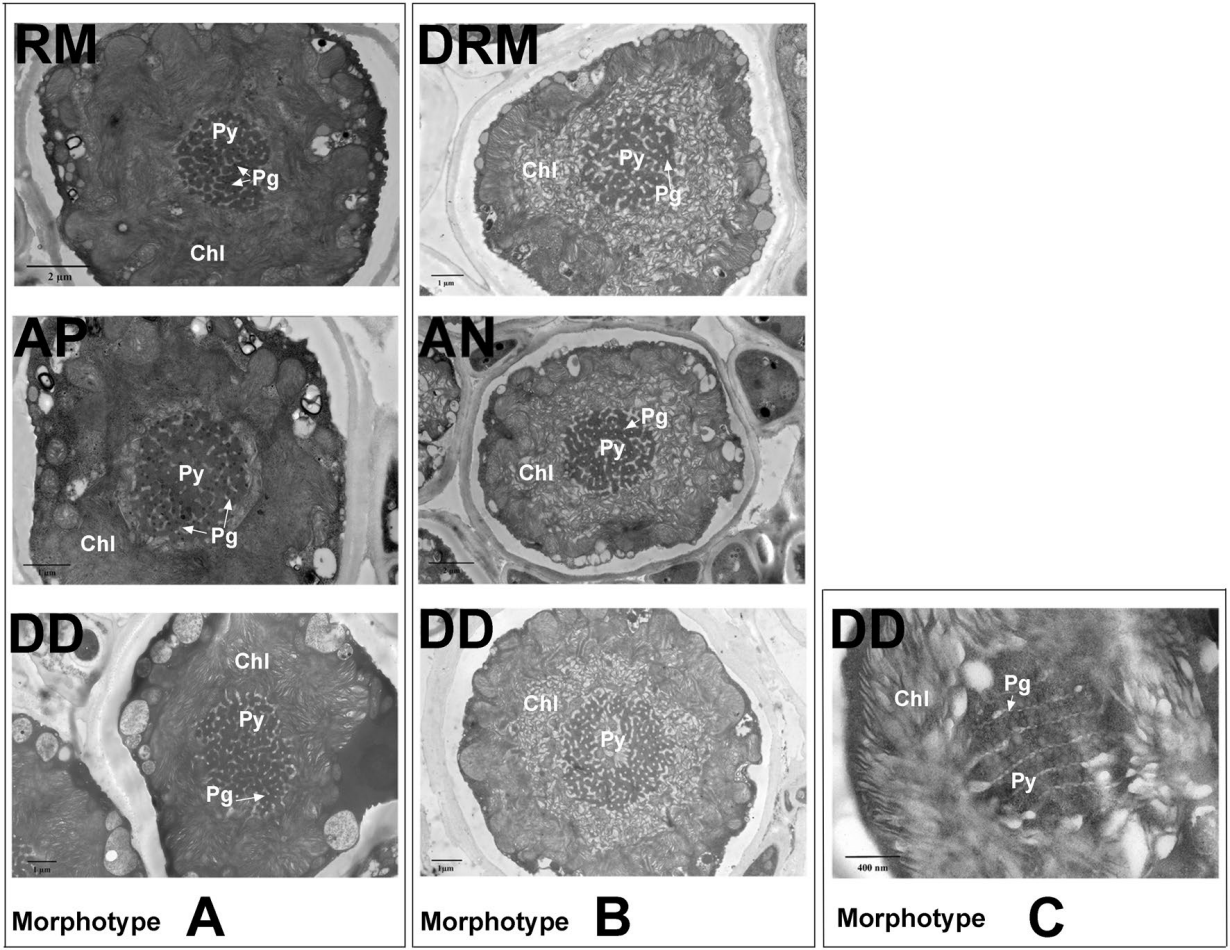
Cross-sections of the crustose lichens from Fuentidueña de Tajo obtained by TEM. Morphotype A of phycobionts found in *Diploschistes diacapsis* (DD), *Acarospora placodiiformis* (AP) and *Rhizocarpon malenconianum* (RM). Morphotype B of phycobionts found in *Diploschistes diacapsis* (DD), *Acarospora nodulosa* (AN) and *Diplotomma rivas-martinezii* (DRM). Morphotype C of phycobionts found in *Diploschistes diacapsis* (DD). *Py* pyrenoid; *Pg* Pyrenoglobuli, *Chl* Chloroplast. Bars: 400 nm, 1 μm, and 2 μm.

Phycobionts identified in five selected specimens (one per species) via TEM were also analysed by Sanger sequencing of the nrITS region. Thalli from DD, AN, RM and DRM showed unsuccessful Sanger sequences, and only AP showed a unique sequence that matched *T*. *cretacea* (included in the phylogeny: Fig. 2). TEM analyses of phycobionts distinguished two predominantly *Trebouxia* morphotypes based on ultrastructural features of the pyrenoids (Py) and plastids (Chl)^54^. The morphological characteristics of each morphotype can be observed in detail in Fig. 4, and more cells of each type can be observed in Supplementary Fig. S1. Morphotype A found in DD, AP and RM cells (Fig. 4) showed a single central Py related to the *gigantea* type described by Friedl^54^, with pyrenoglobuli (Pg) uniformly distributed within the Py matrix and a dense thylakoid membrane arrangement. Morphotype B was found in DD, AN and DRM and showed the same *gigantea* Py type but with lax ordering of thylakoid membranes (Fig. 4). Only in the DD thalli did we find both morphotypes A and B and a few cells showing morphotype C with a Py related to the *gelatinosa* type (Fig. 4; morphotype C).

### Mineralogical and macro- and micro-element patterns in both locations

Mineralogical patterns of the studied samples (FU1 to FU3 and TI1 to TI3) are shown in Supplementary Fig. S2 and Supplementary Table S1, including the quantitative estimates of the identified phases. The mineral assemblages of the studied substrates consist mainly of gypsum, calcite and quartz, with minor contents of sheet silicates (illitte, kaolinite and chlorite) and feldspars (orthoclase and anorthite), and also include calcium oxalates, whewellite (calcium oxalate monohydrate), and weddellite (calcium oxalate dihydrate), which are concentrated in the outer rim of the substrates. Gypsum soils from the two locations showed similar mineralogical assemblages.

The micro- and macro-element contents in soil samples collected from FU and TI are shown in Supplementary Fig. S3. The patterns of micro- and macro-elements found in the two locations were very similar: the most abundant micro-elements were Fe–Sr–Si and Ti, with values ranging from 4,484.78 to 85.54 in TI, and the remaining elements had values ranging from < 0.01 to 33.65. Fe and Sr (the most abundant elements in both locations) showed higher values in FU than in TI.

### Mycobiont barcode identification and nucleotide diversity

To ensure the correct lichen identification, the mycobiont nrITS of each specimen included in the study was analysed. The datasets for DD from both locations were grouped into a clade (- BS/56 PP) with three sequences deposited in GenBank from Fernández-Brime et al.^55^ (Supplementary Fig. S4). AN and AP sequences from both locations also formed a well-supported clade (97 BS/99 PP and 100 BS/100 PP), including sequences previously reported for these two lichen species^56^ (Supplementary Fig. S5), and RM from FU and TI formed a new, well-supported clade (100 BS/100 PP) compared with that in the previously published phylogeny for this genus^57^ (Supplementary Fig. S6). The new DRM sequences from FU formed a well-supported clade (97 BS/100 PP), including the DRM sequences downloaded from Molina et al.^58^ (Supplementary Fig. S7).

## Discussion

The present study expands knowledge concerning the interactions among crustose lichenized and lichenicolous lichens growing on gypsum outcrops in the central Iberian Peninsula with a low-dry ombroclimate^59^.

Sanger results for the crustose lichen species included in the present study indicated no apparent selectivity (generalists) of microalgae, as they associated with four different *Trebouxia* species. *Diploschistes diacapsis*, as a donor lichen, was able to associate with at least three *Trebouxia* spp. *R. malenconianum*, as an obligate lichenicolous lichen, harboured two of them, using the strategy of maintaining the algal host. *Acarospora nodulosa* and *A. placodiiformis* maintained the same three *Trebouxia* spp. from their host (*D. diacapsis*) but also associated with another species, *Trebouxia* sp. OTU A25, a strategy that could be considered algal switching. Therefore, AN and AP seem to use both types of strategies to acquire phycobionts. In *D. rivas-martinezii* (non-parasitic), we detected only two different *Trebouxia.* This result could be influenced by its different lifestyle (epilithic) with respect to that of *D. diacapsis* or by the small number of specimens analysed.

The present study shows, through 454-pyrosequencing, that *D. diacapsis*, considered the phycobiont donor, harbours the four previously mentioned *Trebouxia* species, which seem to be completely transferred to *R. malenconianum*. *A. nodulosa* thalli maintain part of this *Trebouxia* lichen guild and incorporate and integrate compatible new strains or species in a pre-existing thallus^39^, such as *Myrmecia israeliensis*. Even though these results should be interpreted with caution due to the small number of specimens analysed, our study reveals that including metabarcoding analyses could be key to understanding the phycobiont relationship in these systems. Photobiont switching seems to be a ubiquitous phenomenon in lichens and appears to play a vital role in lichen adaptation to environmental conditions^46,47,60–64^. Association with a wide range of symbionts may help lichens survive under harsh environmental conditions^65–67^, such as those characterizing gypsum biocrusts in a low-dry ombroclimate. Future analyses with Illumina metabarcoding of specimens in different transitional states, including the soil surface directly in contact with the thallus, will help clarify these interchanges.

For decades, studies dealing with phycobiont diversity in lichen thalli were primarily based on Sanger sequencing. In recent years, metabarcoding analyses have uncovered substantial species diversity associated with lichen symbioses^4,68–71^ because HTS techniques detected a vast number of genotypes that would otherwise have remained undetected using conventional PCR amplifications. In fact, the discovery of phycobiont multiplicity within a single lichen thallus^38,39,41,72^ and the exclusive use of Sanger sequencing in the majority of studies led the lichenologist community to consider whether terms such as specificity and selectivity (related to symbiotic association patterns) should be revised under this new perspective. In Paul et al.^73^, the potential of Sanger sequencing and HTS metabarcoding to reveal phycobiont diversity was compared in *Lasallia hispanica* and *L. pustulata*, and it was determined that Sanger technology consistently yielded the most phycobiont sequences in each sample. However, if the second most abundant microalga exceeded 30% of the total HTS reads in a sample, Sanger sequencing generally failed and generated ambiguous Sanger sequences showing double peaks. In this study, all the species selected showed ambiguous Sanger sequence frequencies ranging from 33% in *R. malenconianum* to 71% in the case of *A. nodulosa* and *D. rivas-martinezii* (Supplementary Fig. S4). HTS performed on *D. diacapsis* and *A. nodulosa* indicated that the abundance of the second phycobiont exceeded 30% (Table 1), so we assume that these ambiguous Sanger sequences reflect a high percentage of the specimens showing algal multiplicity.

To confirm whether the presence of double peaks indicated the presence of more than one phycobiont, four thalli each of *D. diacapsis*, *A. nodulosa* and *A. placodiiformis*, which showed unsuccessful Sanger sequences for the nrITS region, were selected to amplify a second genetic marker (LSU DNA). All the specimens analysed showed double peaks at both genetic markers (data not shown). Several authors reported that sequences showing double peaks, or polymorphic sequences, in electrophoretograms were removed from their analyses^41,44,53^; perhaps those removed sequences constituted specimens showing algal coexistence in these lichen species, which were not taken into account. The ability of Sanger sequencing to address a wide variety of inquiries in lichen ecology and evolution is not in question, but it is important to point out that phycobiont coexistence could vary depending on the lichen species and could be the general rule in some lichen species^37,38,46–48,74^. We hypothesized that coexistence could be related to, or favoured by, specific thallus architectures, such as the crustose type, and this factor should be considered when studies about patterns of fungal-algal associations are carried out.

As mentioned in the introduction, this work is complementary to that of Moya et al.^49,50^. These studies revealed the associations between *Asterochloris mediterranea* and *Cladonia* spp. and between *Myrmecia israeliensis* and squamulose lichens located in gypsum BSCs. Therefore, we can conclude that three microalgal genera (*Trebouxia*, *Asterochloris* and *Myrmecia*) are available in these Iberian gypsum biocrusts, but multidirectional selection is performed by the mycobiont/phycobionts. In terms of local scale specificity, foliose and squamulose species can be regarded as specialists (foliose/*Asterochloris mediterranea* and squamulose/*Myrmecia israeliensis*); in contrast, the crustose species included in this study are considered generalists.

These results raise the question of which factors might influence the above-suggested selection, due to the peculiarity of the gypsum biocrust mineralogical and macro- and micro-element patterns were analysed to evaluate their influence on the microalgal pool available in the substrate. Preliminary results for mineralogical components showed gypsum, calcite, sheet silicates and feldspars which are usual constituents of the gypsiferous rocks from FU and TI. Although Fe and Sr were the most abundant microelements in the substrates and no significant relationship was found with the microalgal diversity, we hypothesize that both elements could influence both the microalgal pool available in the substrate and the presence of particular lichen species^75–82^. Kakeh et al.^83^ also encountered a significantly higher content of Fe under bioencrusted soils compared to uncrusted soils, and Ochoa-Hueso et al.^84^ positively associated the presence of Fe in the substrate with lichen cover. De los Ríos et al.^28^ suggested that iron minerals are more resistant to lichen action than the other minerals present due to the presence of a rich zone in iron in direct contact with the lichens but not inside the thalli. However, other elements, such as Sr, are known to negatively affect either the abundance or the diversity of algae^85^. Mei et al.^86^ demonstrated that Sr led to a decrease in chlorophyll content (Chl a, Chl b, and total chlorophyll) in *Platymonas subcordiformis* (Volvocaceae). It was also suggested that the substrate determines the sets of cyanobionts available for sharing among mycobionts of terrestrial and epiphytic lichen guilds^87^. However, due to possible implications for phycobiont availability and the influence of given elements on algal physiology, we believe that the analysis of gypsum soil chemical properties should not be neglected when lichen microalgal diversity is investigated, and it must be complemented with HTS studies to check the microalgal pool available at the sites.

As we suggested before, thallus architecture could be related to association patterns and the presence of some specific algae, but lichen morphology and development have usually been studied from a descriptive or ecophysiological point of view^88–91^, and little information is available related to these factors. However, Souza-Egipsy et al.^92,93^ analysed the ultrastructural and biogeochemical features of different substrates (gypsum, volcanic rock and sedimentary rock) to explore the relationships between lichen symbiosis and the lichen–soil interface and revealed that thallus morphology is key in conditioning contact with the surface. Our results suggest a correlation between symbiotic patterns and lifestyle/architecture (foliose/*Asterochloris mediterranea*; squamulose/*Myrmecia israeliensis* and crustose/*Trebouxia* spp.) and suggest that the morphology and/or growth type of each mycobiont may influence its selection of a particular microalgal genus. The results obtained here should also be taken into consideration when designing and managing conservation plans to protect these singular and vulnerable ecosystem types.

## Methods

### Sampling

Two representative locations in the upper mesomediterranean low-dry area of Madrid (Spain) with gypsum BSCs were selected: Fuentidueña de Tajo (FU) (40° 07′ 41" N, 03° 09′ 14" W/571 m a.s.l.) and Ti-tulcia (TI) (40° 07′ 32" N, 03° 33′ 15" W/521 m a.s.l.).

**Figure 5:**
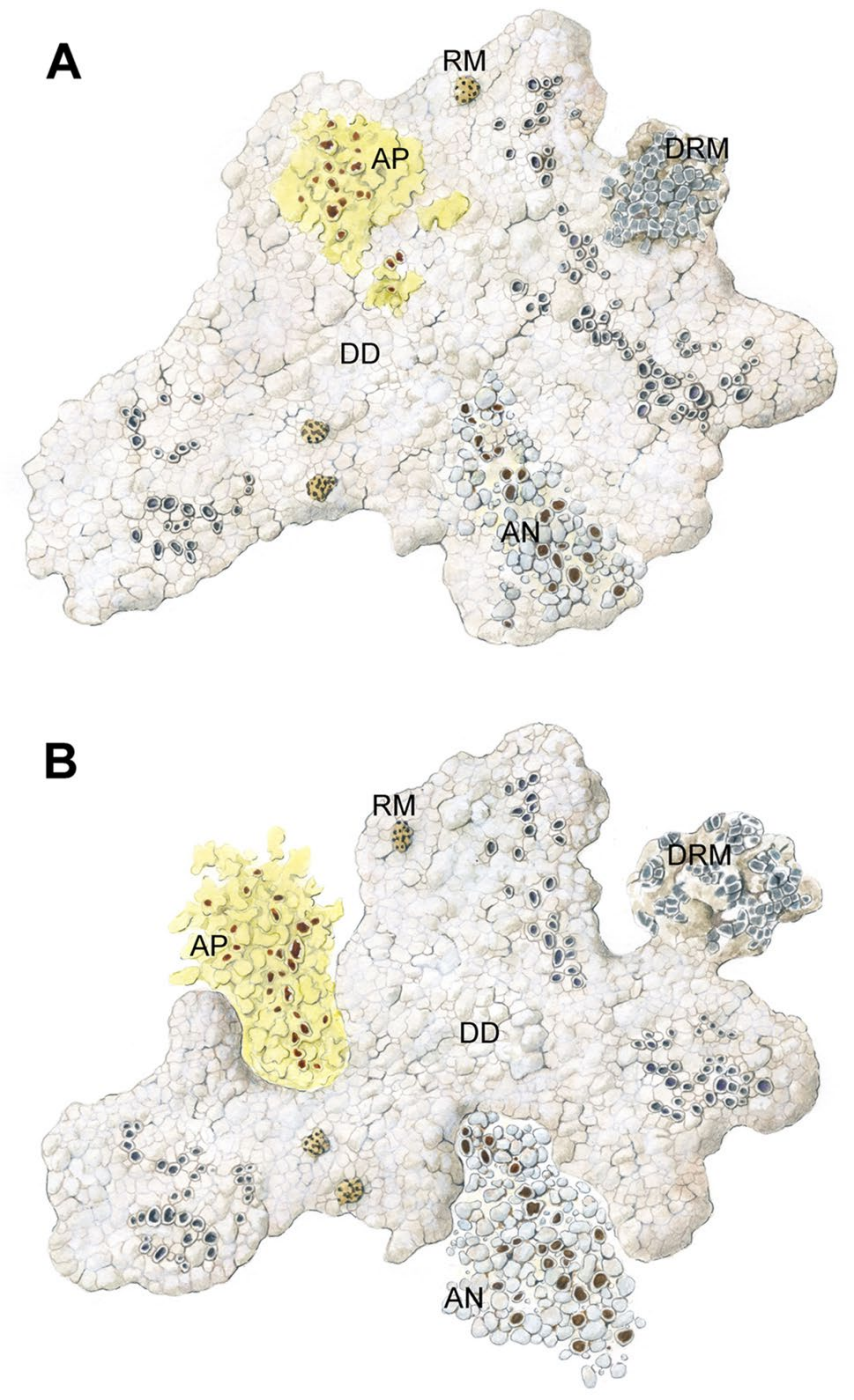
Depiction of the crustose lichen communities composed of *Diploschistes diacapsis* (DD), *Acarospora nodulosa* (AN), *Acarospora placodiiformis* (AP), *Rhizocarpon malenconianum* (RM) and *Diplotomma rivas-martinezii* (DRM)*.* During their first growth stages, *A. nodulosa* and *A. placodiiformis* are parasites of *D. diacapsis* (Fig. 5A), while in mature stages, they develop independent thalli (Fig. 5B). *Rhizocarpon malenconianum* is an obligate lichenicolous lichen on *D. diacapsis* hosts, and the epilithic species *D. rivas-martinezii* occurs physically close to *D. diacapsis.*

In these locations, five crustose lichen species were selected due to their peculiar lifestyles as facultative or obligate lichenicolous lichens: *A. nodulosa* (AN) and *A. placodiiformis* (AP) are parasites of *D. diacapsis* (DD) during their first growth stages (Fig. 5A parasitic state, Supplementary Fig. S8A, and C), while in mature stages, they are able to develop independently, becoming autonomous thalli (Fig. 5B, Supplementary Fig. S9B and D). *Rhizocarpon malenconianum* (RM) is an obligate lichenicolous lichen on *D. diacapsis*^12^ (Fig. 5 A, B, Supplementary Fig. S8E). The epilithic species *D. rivas*-*martinezii* (DRM) is regularly found physically close to *D. diacapsis*^11^ (Fig. 5 A, B, Supplementary Fig. S8F).

A total of 161 thalli were collected from both locations: 55 autonomous specimens of *D. diacapsis* (DD), 42 of *A. nodulosa* (AN) and 51 of *A. placodiiformis* (AP). We also included *R. malenconianum* (RM; n = 6) (only detected in Fuentidueña) and *D. rivas*-*martinezii* (DRM; n = 7) when they occurred at the sites. The samples were dried and stored at − 20 °C until processing. Each lichen specimen used in this study was given a species_number and sample_location code, i.e., DD 4 TI.

### DNA extraction, PCR amplification and Sanger sequencing

Lichen thalli were examined under a stereomicroscope to remove soil particles and were immersed sequentially in ethanol and NaOCl^94^ to remove surface contaminants and to ensure the intrathalline origin of the sequenced microalgae. Fragments from different parts of each thallus were randomly excised and pooled together. The mycobiont and the primary phycobiont were identified by Sanger sequencing. Total genomic DNA from all the samples was isolated and purified using the DNEASY PLANT MINI KIT (Qiagen, Hilden, Germany) following the manufacturer’s instructions. The phycobiont locus encoding the nrITS region was amplified using the primer pair nr-SSU-1780^22^ and ITS4^95^. The fungal nrITS region was amplified using the primer pair ITS1F^96^ and ITS4^95^. Both mycobiont and phycobiont PCRs were performed as described in Molins et al.^46,72^. The PCR products were visualized on 2% agarose gels and purified using the GEL BAND PURIFICATION KIT (GE Healthcare Life Science, Buckinghamshire, England). The amplified PCR products were sequenced with an ABI 3100 Genetic Analyzer using the ABI BIG-DYE TERMINATOR CYCLE SEQUENCING READY REACTION KIT (Applied Biosystems, Foster City, California). Sanger sequences were visualized and manually evaluated with Chromas v 2.6.6.0 (https://technelysium.com.au/wp/chromas/). Double peaks were identified as peaks with a secondary peak height 25% above the maximal peak height. We excluded sequences with ambiguous base calls and/or underlying peaks in the electrophoretogram that could not be resolved. Since it was impossible to perform robust taxonomic assignment for these sequences, we excluded them from the dataset, and we noted them as unsuccessful Sanger sequences. All sequences passing the quality filter were considered unique, showing only one predominant phycobiont.

### 454-pyrosequencing analyses

One additional thallus of DD collected in Fuentidueña, containing AN and RM in their parasitic state (Supplementary Fig. S9), was pyrosequenced following the protocol described in Moya et al.^38^ and Molins et al.^72^. The numbers of cycles of PCR I and PCR II were determined by the average Ct (cycle threshold) of RT-PCR I (DD = 20, AN = 17, and RM = 15) and RT-PCR II (DD = 6, AN = 7, and RM = 7). Algal nrITS sequences were determined using a GS Junior 454 system (Roche 454 Life Sciences, Branford, CT, USA) following the Roche Amplicon Lib-L protocol at the Genomics Core Facility, SCSIE, University of Valencia (Spain). Raw read datasets obtained from these thalli were individually trimmed as described in Moya et al.^38^. The amplicon sequences from each SFF file were extracted using the sff_extract script (https://bioinf.comav.upv.es/sff_extract/index.html) to generate fasta and quality files that were combined into a single fastq file with a custom Python script. The fastq file was examined with FastQC software v 0.10.1^97^ to inspect the length and quality of the reads. Processing and trimming of the low-quality ends of the sequences were performed with the program Trim_Edges from the seq_crumbs-0.1.8 package based on the graphs generated with FastQC software. Eighteen nucleotides from the 5′ ends (sequencing primer and MID sequences) and 22 nucleotides from the 3′ ends (low-quality nucleotides) were removed. Sequences shorter than the final sequence alignment were not considered in the analyses. The resulting fasta file from each library was clustered with BLASTCLUST software v 2.2.26^98^ based on a 99% score coverage threshold and a 90% length coverage threshold (S 99-L 0.9). Each OTU (operational taxonomic unit) obtained from the output clustering list file was converted into an individual fasta file using a custom Python script that included the removal of unique sequences (singletons). The different sequences from each OTU were aligned by MUSCLE^99^ and manually adjusted using MEGA v 5.0^100^, and a consensus sequence file was manually generated for each OTU and labelled with the lichen code followed by the species_number of the OTU, i.e., DD OTU 1. These consensus sequences of the OTUs were classified into genera (*Trebouxia*, *Asterochloris* or any of the additional algal genera) using BLAST searches^98^ against the GenBank sequence database^101^ and were labelled with the lichen code followed by the species_number of the OTU, i.e., DD OTU 1.

### Phycobiont phylogenetic analysis

A multiple alignment of nrITS sequences was prepared, including the phycobiont sequences obtained by Sanger sequencing (Supplementary Table S2), the consensus sequence OTUs obtained by 454-pyrosequencing analysis (Supplementary Table S2) and 51 selected OTU sequences from Clade A described by Muggia et al.^102^. We included *Asterochloris erici* (AF345439) as an outgroup. The alignment was carried out with MAFFT v 7.0^103,104^ using default parameters. The resulting alignment was manually optimized to replace gaps at the ends of OTUs from^102^ with an IUPAC base representing any base (N). The multiple sequence alignment was processed in Gblocks v. 0.91b^105^ with the least stringent parameters. This software allows for automatically removing conflicting regions in the alignment. Optimal substitution models for the two partitions within the nrITS region (ITS1+2, 5.8S) were inferred using jModelTest v 2.0^106^ and by applying the Akaike information criterion^107^. This analysis favoured the GTR + G model for the ITS1 + ITS2 partition, and TrNef for the 5.8S. maximum likelihood (ML) analysis was implemented in RAxML v 8.1.11^108^ using the GTRGAMMA substitution model for the two delimited nrITS partitions. Bootstrap support (BS) was calculated based on 1,000 pseudoreplicates^109^. Bayesian inference (BI) was carried out in MrBayes v 3.2^110^. Settings included two parallel runs with six chains over 20 million generations starting with a random tree and sampling after every 200th step. We discarded the first 25% of the data as burn-in, and the corresponding posterior probabilities (PPs) were calculated from the remaining trees. Estimated sampled sized (EES) values above 200 and potential scale reduction factor (PSRF) values approaching 1.000 were considered indicators of chain convergence. All analyses were implemented with the CIPRES Science Gateway v 3.3^111^. Phylogenetic trees were visualized in FigTree v 1.4.1^112^.

### Microscopic examinations

The ultrastructure of the phycobionts was characterized by transmission electron microscopy (TEM) from five thalli (one specimen per species). Specimens from AN, AP, RM and DRM were sampled in a parasitic state from the same DD thallus (Fig. 5A), and their primary phycobionts were identified by Sanger sequencing. For TEM, the cells were fixed and dehydrated as described in Molins et al.^46,72^. Samples were embedded in Spurr’s resin according to the manufacturer’s instructions. Sections (90 nm) were cut and mounted as described in Moya et al.^38^. The sections were observed with a JEOL JEM-1010 (80 kV) electron microscope equipped with a MegaView III digital camera and ‘AnalySIS’ image acquisition software. TEM examinations were carried out at the SCSIE Service of the University of Valencia.

### X-ray powder diffraction mineralogical analysis

X-ray powder diffraction (XRD) was performed to investigate the mineralogical composition of three samples from FU (FU1–FU2–FU3) and three from TI (TI1–TI2–TI3). The FU1 and TI1 samples were collected from the soil immediately below the corresponding biocrust (0–0.5 cm); the FU2 and TI2 samples spanned a depth of approximately 2–5 cm, and the FU3 and TI3 samples were obtained as a mixture of the corresponding FU1 + FU2 and TI1 + TI2 samples. Dried samples were ground into a powder in a Fritz Pulverisette P9 rotor mill until they could pass through a 230 ASTM sieve. Random powders were obtained using the Niskanen^113^ method, and oriented aggregates of fractions were used for the identification of sheet silicates according to the Pansu and Gautheyrou^114^ method. The collection of XRD data was performed using a Bruker D8 instrument with the Diffrac Plus System, Cu-Kα radiation, and a beam voltage and a current of 40 kV and 20 mA, respectively; a Ni filter, a step size of 0.03°2θ, and a step time of 96 s were used. The EVA program in combination with the ICDD database was used for data evaluation following the Warshaw and Roy^115^ method for the identification of sheet silicates. Semi-quantitative estimates of the mineral phases were carried out according to Davis et al.^116^. XRD analysis was performed at the X-ray powder laboratory of the SCSIE Service of the University of Valencia.

### Determination of macro- and micro-elements in gypsum samples

Macro- and micro-element contents were determined according to ISO 11.885^117^ by inductively coupled plasma optical emission spectrometry (ICP-OES) using Thermo ICAP 6500 Duo equipment (Thermo Fisher Scientific, Waltham, MA, USA). In a microwave furnace, 200 mg of each sample was added to a 25 ml tube with a mixture of 4 ml of HNO_3_ (68% purity) and 1 ml of H_2_O_2_ (33% purity) for subsequent digestion. Three hundred millilitres of high-purity de-ionized water, 30 ml of H_2_O_2_ (33% purity) and 2 ml of H_2_SO_4_ (98% purity) were also added to the Teflon reactor. The microwave heating digestion programme consisted of 3 steps: starting at 20 °C and 40 bar, increasing 10 bar/min for 30 min up to 220 °C, and remaining at 220 °C for 20 min. After cooling, the mineralized samples were transferred to double gauge tubes (10 ml for the micro-elements and 25 ml for the macro-elements), and the volume was brought up with high-purity de-ionized water. A multi-mineral standard solution containing 31 minerals, supplied by SCP Science (Quebec, Canada), was used to prepare calibration standards in high-purity de-ionized water. For ICP-OES analyses, two control samples containing high-purity de-ionized water and a multi-mineral standard were used. Each mineral determination was performed at specific wavelengths ranging from 167.1 to 670.8 nm. The concentrations of macro- and micro-elements were calculated according to the formula “mg kg^−1^ or μg kg^−1^ = (C x D)/W”, where C is the element concentration, D is the dilution factor and W is the sample weight. These analyses were carried out at the Laboratorio de Ionómica (CEBAS-CSIC).

### Mycobiont phylogenetic analysis

Independent multiple alignments were performed, including alignment of the new mycobiont nrITS sequences generated in this study (Supplementary Table S2) and selected sequences downloaded from GenBank from previously described phylogenies. *Thelotrema* spp. (HQ650717 and AJ508684) were selected as outgroups in the phylogeny constructed for DD, *Pycnora sorophora* (KX132977) was selected for AN/AP, *Fuscidea intercincta* (AF483605) was selected for RM, and *Physconia grisea* (AF542506) was selected for DRM. Each alignment was carried out with MAFFT v 7.0^103,104^ using default parameters. Optimal substitution models for the two partitions within the nrITS (ITS1 + 2, 5.8S) were inferred using jModelTest v 2.0^106^ and by applying the Akaike information criterion^107^. In the case of DD and DRM, this analysis favoured the GTR + G model for the ITS1 + ITS2 partition, K80 for the 5.8S partition, GTR + I + G for the ITS1 + ITS2 partition, F81 for the 5.8S partition in *Acarospora* spp., GTR + I + G for the ITS1 + ITS2 partition, and SYM + I + G for the 5.8S partition in RM. Maximum likelihood (ML) analysis was implemented in RAxML v 8.1.11^108^ using the GTRGAMMA substitution model for the two delimited nrITS partitions. Bootstrap support (BS) was calculated based on 1,000 pseudoreplicates^109^. Bayesian inference (BI) was carried out in MrBayes v 3.2^110^. Settings included two parallel runs with six chains over 20 million generations starting with a random tree and sampling after every 200th step. We discarded the first 25% of the data as burn-in, and the corresponding posterior probabilities (PPs) were calculated from the remaining trees. Estimated sampled sized (EES) values above 200 and potential scale reduction factor (PSRF) values approaching 1.000 were considered indicators of chain convergence. All analyses were implemented with the CIPRES Science Gateway v 3.3^111^. Phylogenetic trees were visualized in FigTree v 1.4.1^112^.

## Supplementary information

Supplementary Figures.

Supplementary Table S1.

Supplementary Table S2.

## Data Availability

Supplementary Tables S2.
