# Supplementary material for: Symbiotic microalgal diversity within lichenicolous lichens and crustose hosts on Iberian Peninsula gypsum biocrusts
Source: Sci Rep. 2020 Aug 20;10:14060. doi: 10.1038/s41598-020-71046-2 (PMC7441164; doi:10.1038/s41598-020-71046-2)

**Supplementary Information**

Authors: Patricia Moya^1*^, Arantzazu Molins^1^, Salvador Chiva^1^, Joaquín Bastida^2^ and Eva Barreno^1^.

**Title: Symbiotic microalgal diversity within lichenicolous lichens and crustose hosts on** **Iberian Peninsula gypsum biocrusts**

1 Botánica, ICBIBE, Fac. CC. Biológicas, Universitat de València, C/ Dr. Moliner, 50. 46100-Burjassot, Valencia, Spain.

2 Geología, Fac. CC. Biológicas, Universitat de València, C/ Dr. Moliner, 50. 46100-Burjassot, Valencia, Spain.

* For correspondence: P. Moya, Phone number: +34 963544376, email: [patricia.moya@uv.es](mailto:patricia.moya@uv.es).

ORCID Patricia Moya 0000-0003-0397-863X

ORCID Arantzazu Molins 0000-0003-3449-1023

ORCID Salvador Chiva 0000-0002-1615-6443

ORCID Joaquín Bastida 0000-0001-8051-6178

ORCID Eva Barreno 0000-0002-2622-4550

Running title: **Microalgal diversity within crustose and lichenicolous lichens**

**Supplementary Figure S1** Cross-sections of morphotype A (A, B and C) and morphotype B (D, E and F). Py: Pyrenoid; Chl: Chloroplast. Bars: 1 μm and 2 μm

**Supplementary Figure S2** Mineralogical composition distribution of soil samples from Fuentidueña de Tajo (FU) and Titulcia (TI) obtained by X-ray powder diffraction

**Supplementary Figure S3** Concentrations (g/100 g and mg/kg) of micro- and macro-elements detected in soil samples collected in Fuentidueña de Tajo (FU) and Titulcia (TI) by ICP OES

**Supplementary Figure S4** Phylogenetic analyses of nrITS mycobionts from 51 thalli of *Diploschistes* *diacapsis.* Values at nodes indicate statistical support estimated by two methods: bootstrap support (BS, RAxML analysis) and posterior probabilities (PPs, MrBayes analysis). The newly obtained sequences were designated by DD and a locality code, Fuentidueña de Tajo (FU) or Titulcia (TI). Accession numbers from sequences retrieved from GenBank accompany each species name, and *Thelotrema* spp*.* were selected as the outgroup. The specimen selected to characterize the ultrastructure of the phycobionts via TEM is indicated in the phylogenetic tree. The specimen selected for the 454-pyrosequencing analyses is indicated in the phylogenetic tree. The scale bar shows the estimated number of substitutions per site.

**Supplementary Figure S5** Phylogenetic analyses of nrITS mycobionts from 51 thalli of *Acarospora placodiiformis* and 43 of *Acarospora nodulosa.* Values at nodes indicate statistical support estimated by two methods: bootstrap support (BS, RAxML analysis) and posterior probabilities (PPs, MrBayes analysis). The newly obtained sequences were designated by AP, AN and a locality code, Fuentidueña de Tajo (FU) or Titulcia (TI). Accession numbers from sequences retrieved from GenBank accompany each species name, and *Pycnora sorophora* was selected as the outgroup. The specimen selected to characterize the ultrastructure of the phycobionts via TEM is indicated in the phylogenetic tree. The specimen of AN selected for the 454-pyrosequencing analyses is indicated in the phylogenetic tree. The scale bar shows the estimated number of substitutions per site.

**Supplementary Figure S6** Phylogenetic analyses of nrITS mycobionts from seven thalli of *Rhizocarpon malenconianum*. Values at nodes indicate statistical support estimated by two methods: bootstrap support (BS, RAxML analysis) and posterior probabilities (PPs, MrBayes analysis). The newly obtained sequences were designated by RM and a locality code, Fuentidueña de Tajo (FU) or Titulcia (TI). Accession numbers from *Rhizocarpon* spp. and *Fuscidea intercincta* sequences retrieved from GenBank accompany each species name. The specimen selected to characterize the ultrastructure of the phycobionts via TEM is indicated in the phylogenetic tree. The specimen selected for the 454-pyrosequencing analyses is indicated in the phylogenetic tree. The scale bar shows the estimated number of substitutions per site.

**Supplementary Figure S7** Phylogenetic analyses of nrITS mycobionts from seven thalli of *Diplotomma rivas-martinezii*. Values at nodes indicate statistical support estimated by two methods: bootstrap support (BS, RAxML analysis) and posterior probabilities (PPs, MrBayes analysis). The newly obtained sequences were designated by DRM and a locality code, Fuentidueña de Tajo (FU). Accession numbers from *Diplotomma* spp., *Diploicia* spp., *Dimelaena* spp. and *Physconia grisea* sequences retrieved from GenBank accompany each species name. The specimen selected to characterize the ultrastructure of the phycobionts via TEM is indicated in the phylogenetic tree. The scale bar shows the estimated number of substitutions per site.

**Supplementary Figure S8** A: *Acarospora nodulosa* parasitic on *Diploschistes diacapsis*, B: autonomous thalli of *A. nodulosa* and *D. diacapsis*, C: *Acarospora placodiiformis* parasitic on *D. diacapsis* during the first growth stages, D: *A. placodiiformis* in mature stages becoming autonomous thalli from *D. diacapsis*, E: *Rhizocarpon malenconianum* as an obligate lichenicolous lichen on *D. diacapsis*, F: epilithic *Diplotomma rivas-martinezii* occurring physically close to *D.* *diacapsis*

**Supplementary Figure S9** Photographs of the three specimens, in their parasitic state, of *Diploschistes diacapsis*, *Acarospora nodulosa* and *Rhizocarpon malenconianum* collected in Fuentidueña and analysed by 454-pyrosequencing

**Supplementary Table S1**. The semiquantitative mineralogical compositions of soil samples from Fuentidueña de Tajo (FU) and Titulcia (TI) determined by X-ray powder diffraction. ICDD file: Number file of the ICDD database. I/Ic: Reference intensity of the compound. SQ: Quantitative estimate (%). RE: Relative error

**Supplementary Table S2**. Phycobiont and mycobiont nrITS GenBank accession numbers for the specimens in this study

**SupplementaryFigureS1**
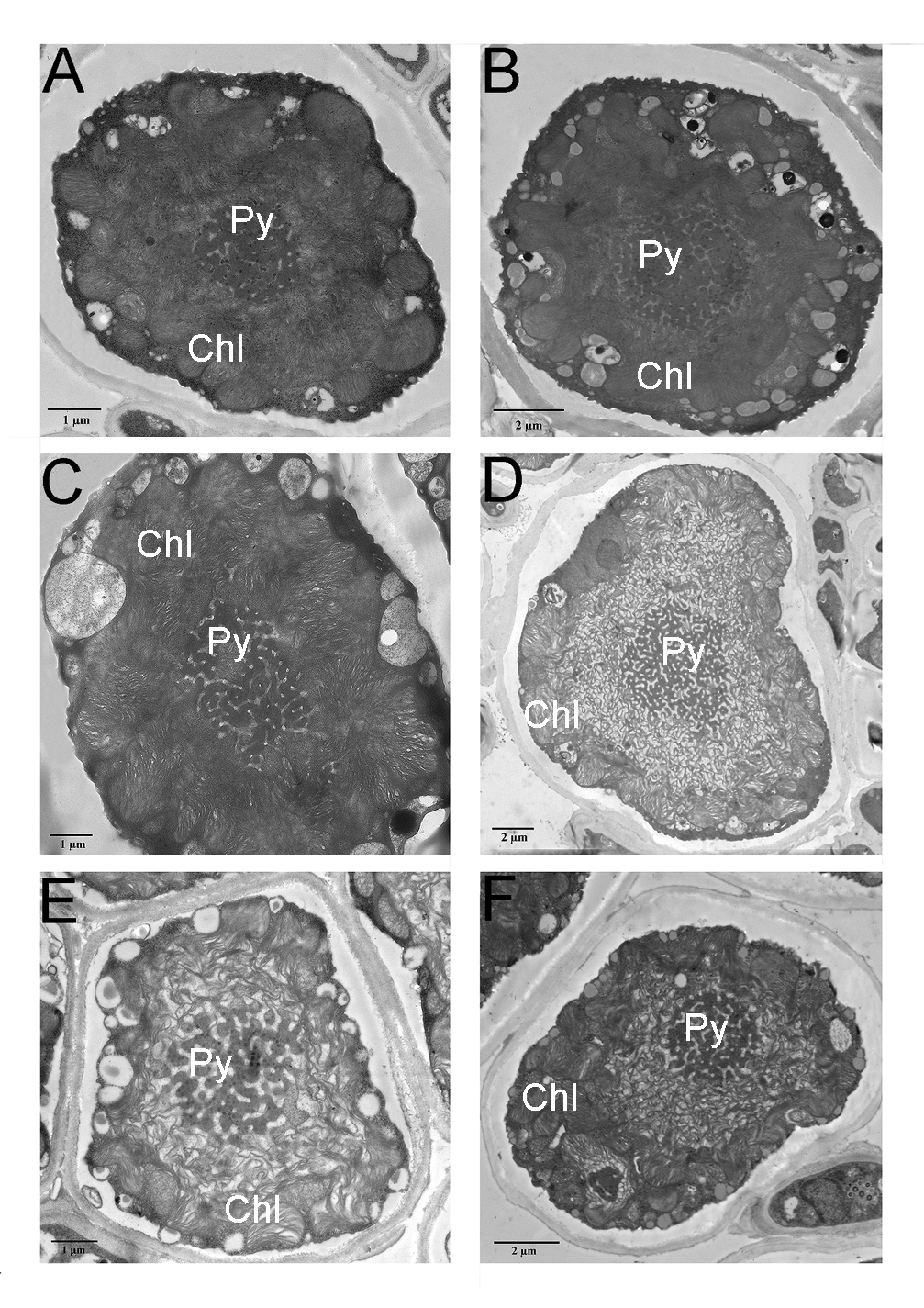


**Supplementary Figure S2**


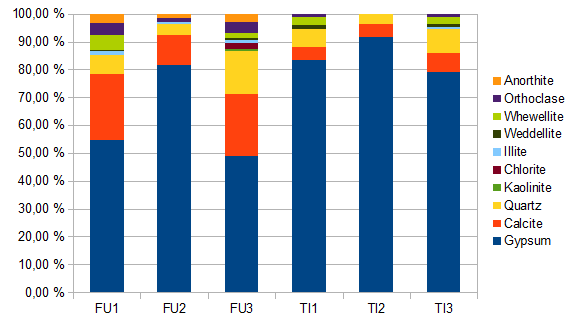
**Supplementary Figure S3**


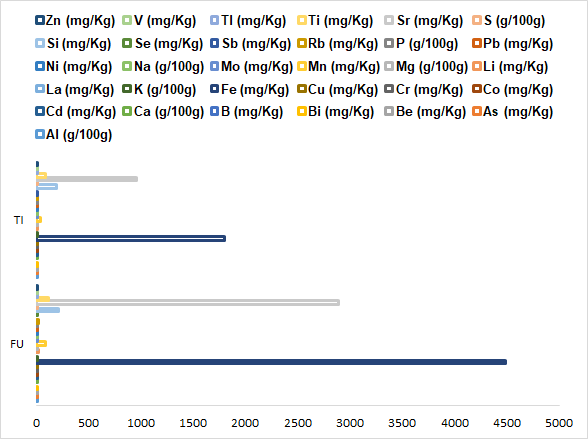


**Supplementary Figure S4**

**
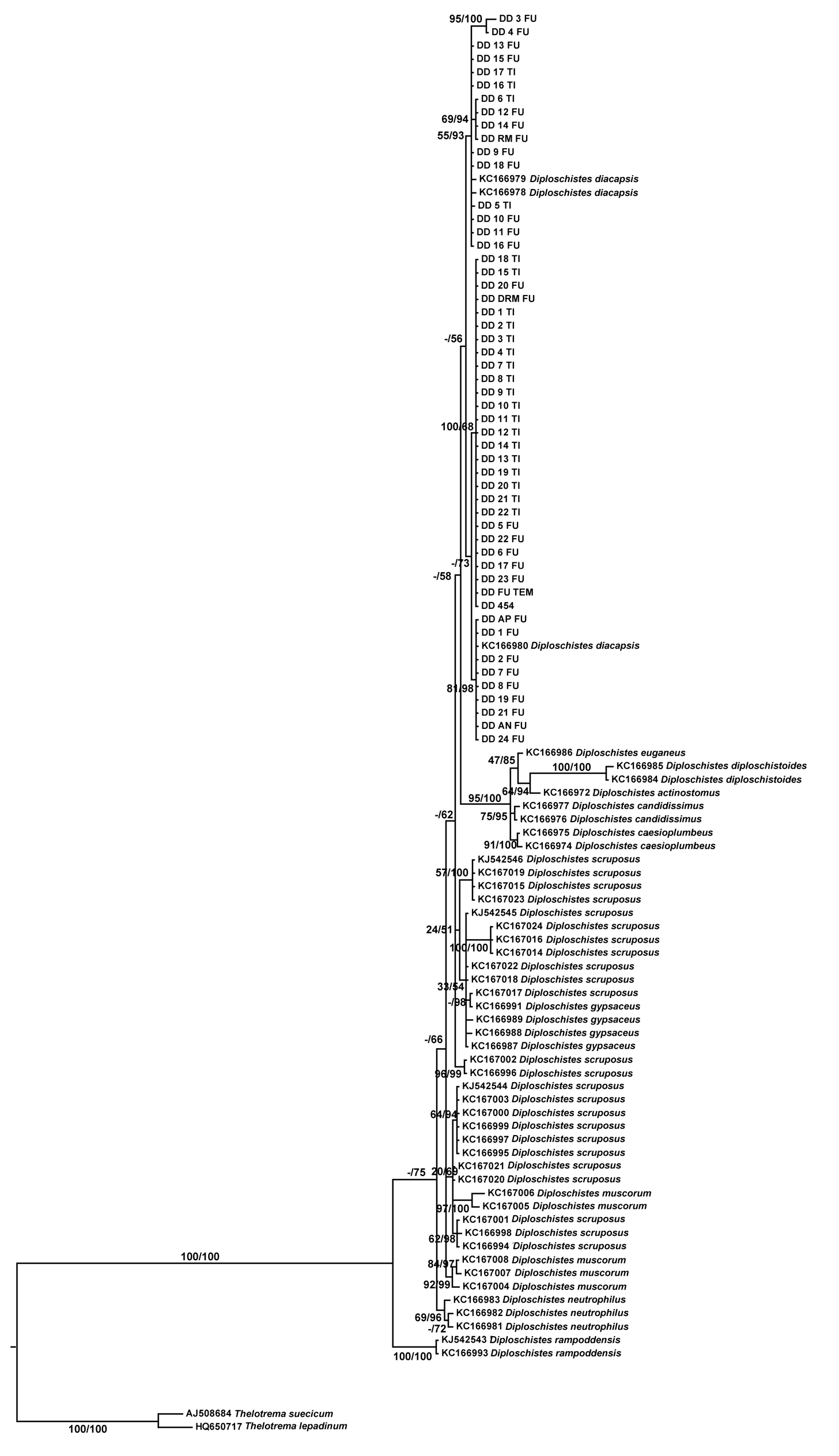
**

**Supplementary Figure S5**


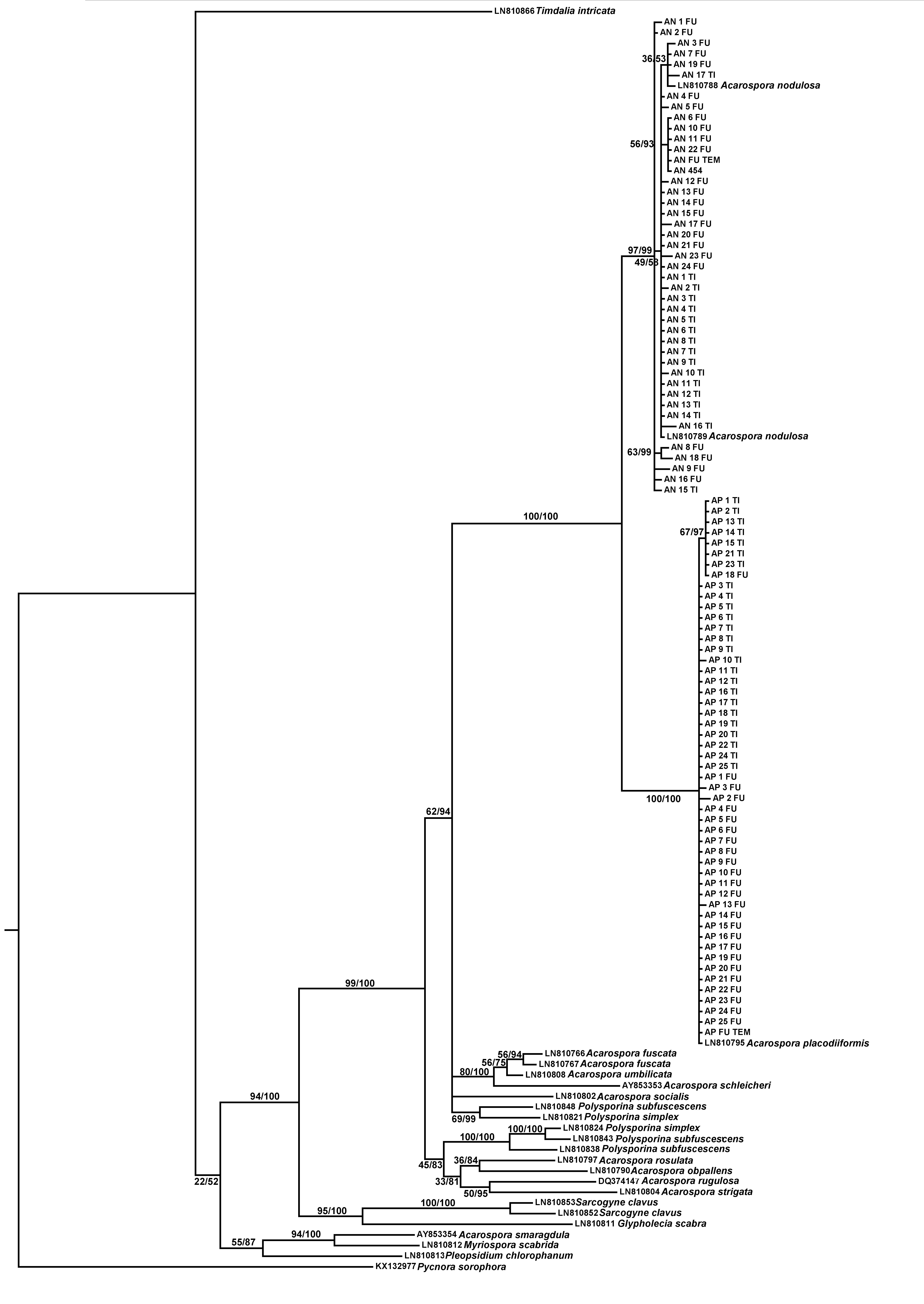


**
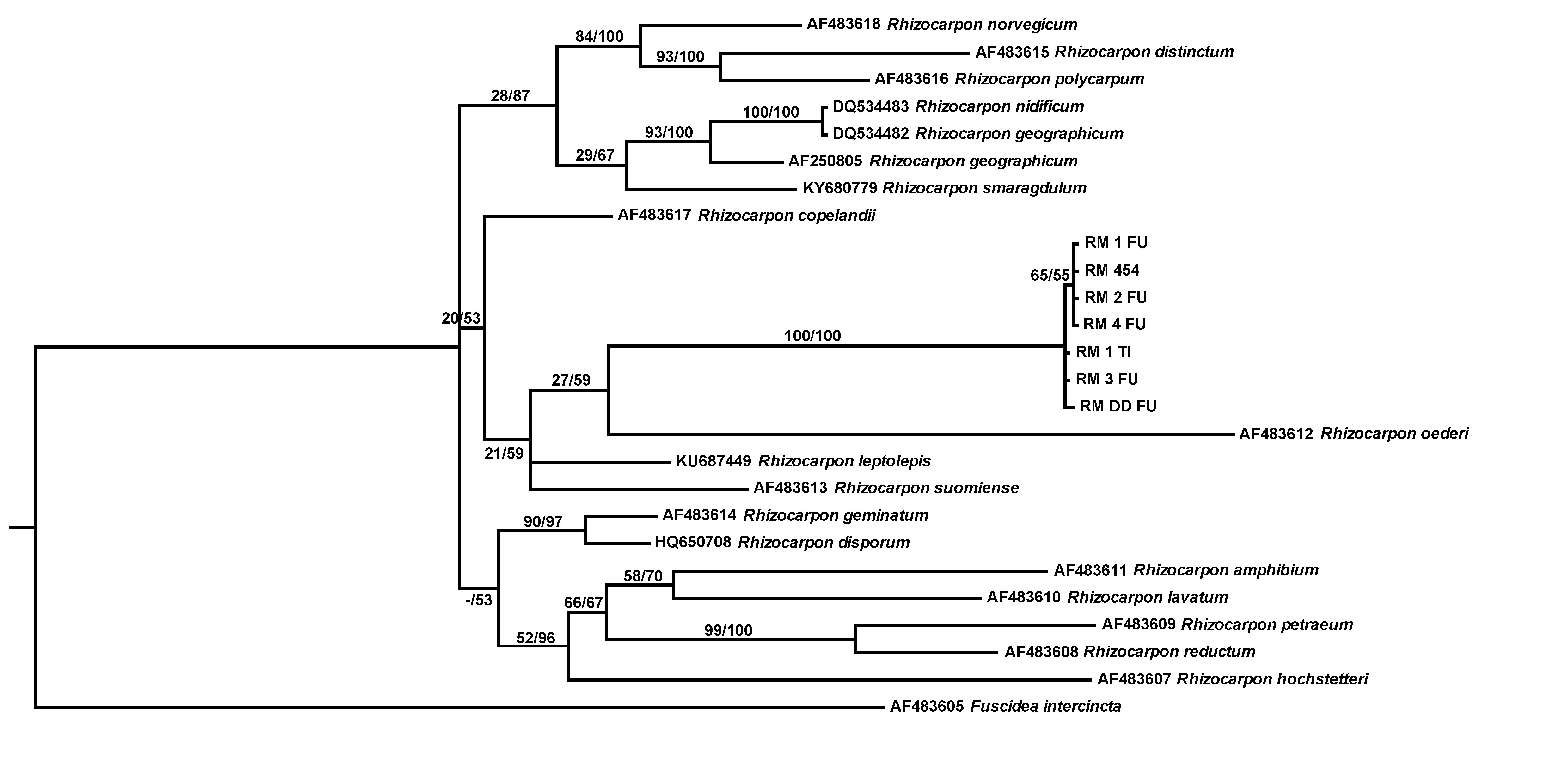
**

**Supplementary Figure S6**


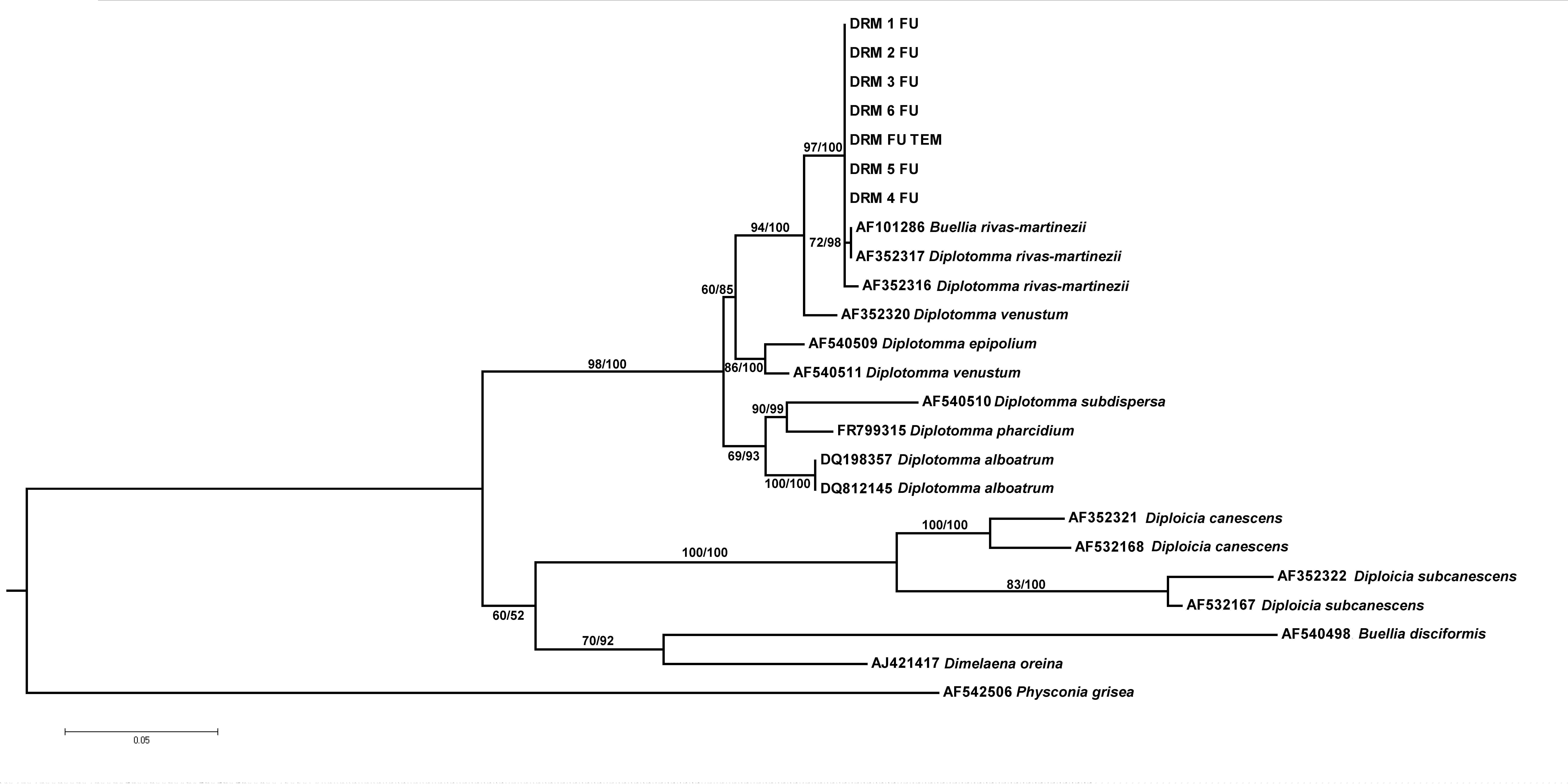


**Supplementary Figure S**7


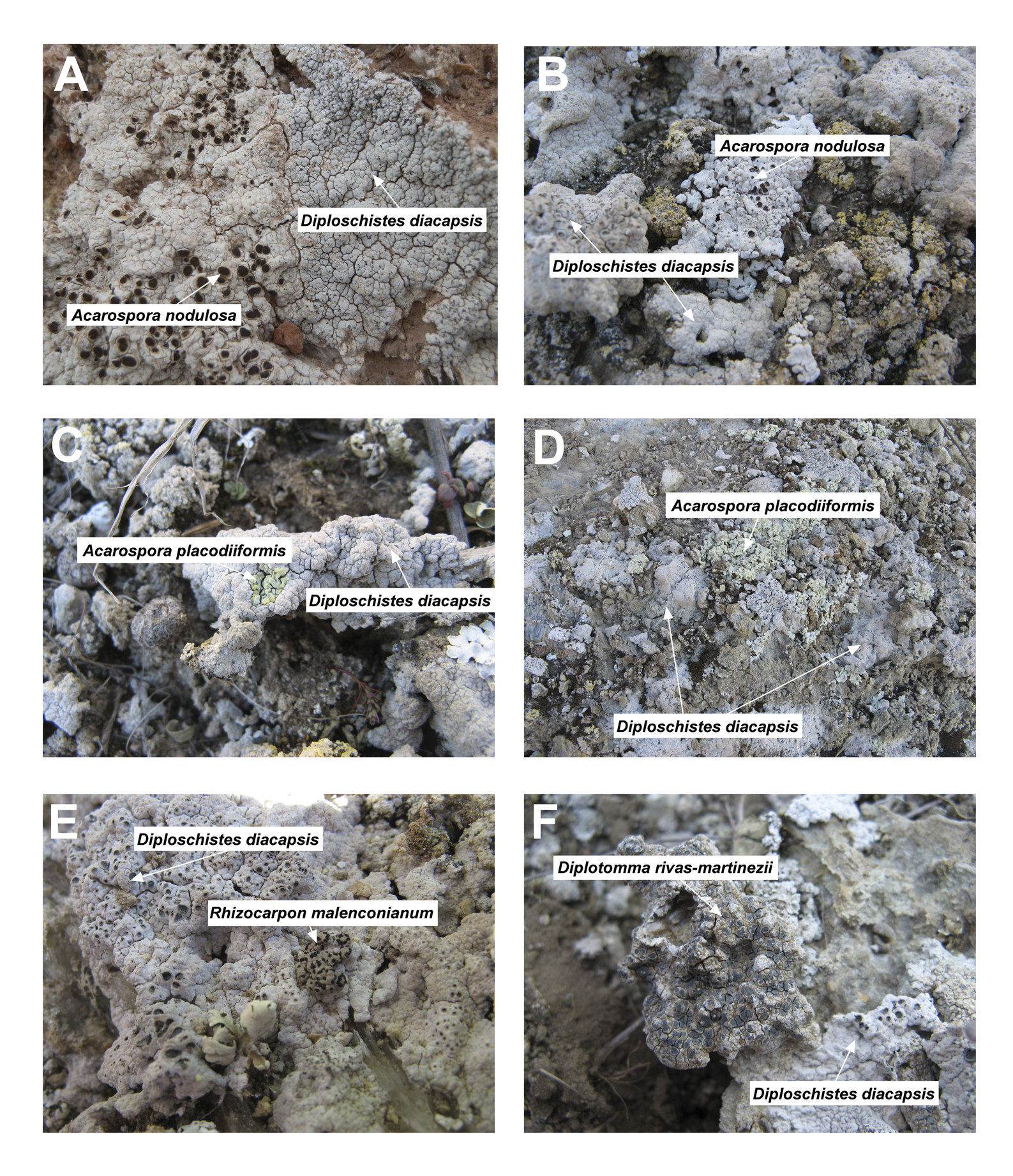


**Supplementary Figure S8**

**Supplementary Figure S9**
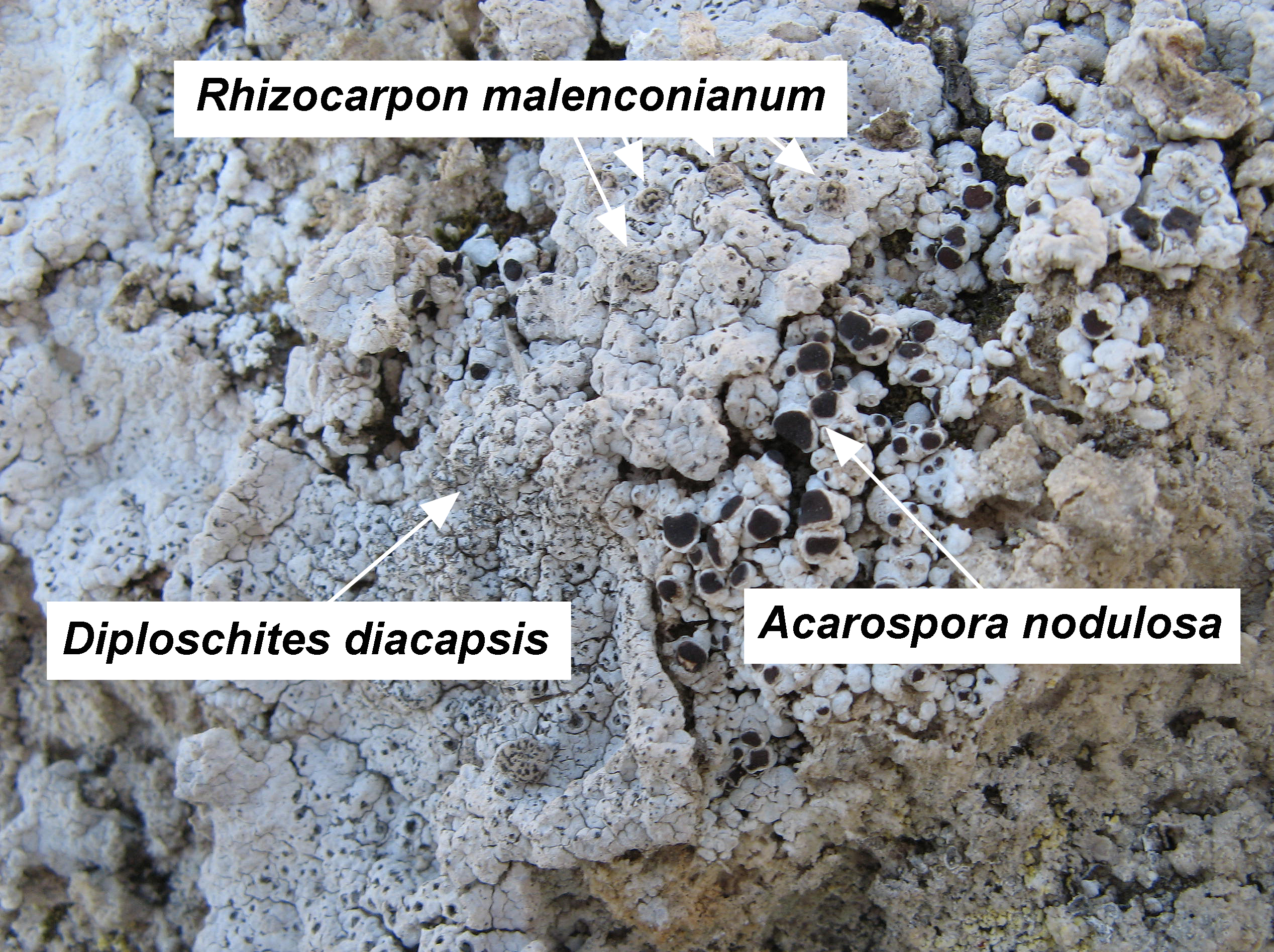

Supplement: Supplementary file 1 — Supplementary Figures. [file 41598_2020_71046_MOESM1_ESM.doc]
