# Supplementary material for: Symbiotic microalgal diversity within lichenicolous lichens and crustose hosts on Iberian Peninsula gypsum biocrusts
Source: Sci Rep. 2020 Aug 20;10:14060. doi: 10.1038/s41598-020-71046-2 (PMC7441164; doi:10.1038/s41598-020-71046-2)
Supplement: Supplementary file 2 — Supplementary Table S1. [file 41598_2020_71046_MOESM2_ESM.docx]

| Sample |  |  |  | FU1 | FU2 | FU3 |  | TI1 | TI2 | TI3 |  |
| --- | --- | --- | --- | --- | --- | --- | --- | --- | --- | --- | --- |
| ICDD file | Compound name | Formula | I/Ic | SQ | SQ | SQ |  | SQ | SQ | SQ | RE |
| 00-036-0432 | Gypsum | CaSO_4_·2H_2_O | 1,7 | 54,6 | 81,5 | 70,7 |  | 83,4 | 91,6 | 78,9 | 0,1 |
| 00-005-0586 | Calcite, syn | CaCO_3_ | 2,0 | 23,9 | 10,8 | 17,3 |  | 4,6 | 4,7 | 7,0 | 0,1 |
| 00-033-1161 | Quartz, syn | SiO_2_ | 3,6 | 6,6 | 3,9 | 6,2 |  | 6,6 | 3,8 | 8,6 | 0,1 |
| 01-083-0971 | Kaolinite | Al_2_(Si_2_O_5_)(OH)_4_ | 1,0 | 0,0 | 0,0 | 0,0 |  | 0,0 | 0,0 | 0,0 | 0,5 |
| 01-078-1997 | Chlorite | (Mg4.715Al.694Fe.269Fe.109Cr.128Ni.011)(Si3.056Al.944)O_10_(OH)_8_ | 0,7 | 0,0 | 0,0 | 0,0 |  | 0,0 | 0,0 | 0,0 | 0,5 |
| 00-002-0056 | Illite | KAl_2_Si_3_Al_4_O_10_ (OH)_2_ | 2,0 | 1,6 | 0,6 | 0,6 |  | 0,0 | 0,0 | 0,8 | 0,5 |
| 01-075-1314 | Weddellite (COD) | CaC_2_O_4_ (H2O) 2.375 | 1,4 | 0,3 | 0,0 | 0,1 |  | 1,2 | 0,0 | 0,9 | 0,5 |
| 01-075-1313 | Whewellite (COM) | CaC_2_O_4_ (H_2_O) | 1,1 | 5,3 | 0,0 | 0,7 |  | 3,0 | 0,0 | 2,6 | 0,5 |
| 01-075-1592 | Orthoclase | KAlSi_3_O_8_ | 0,8 | 4,2 | 1,6 | 2,0 |  | 1,2 | 0,0 | 1,1 | 0,5 |
| 01-078-2330 | Anorthite | Na.25Ca.71 (Al_2_Si_2_O_8_) | 0,5 | 3,5 | 1,5 | 2,3 |  | 0,0 | 0,0 | 0,0 | 0,5 |
|  |  |  |  |  |  |  |  |  |  |  |  |
